# Prenatal Substance Exposure and Neonatal Abstinence Syndrome: State Estimates from the 2016–2020 Transformed Medical Statistical Information System

**DOI:** 10.1007/s10995-023-03670-z

**Published:** 2023-05-23

**Authors:** Kristina D. West, Mir M. Ali, Martin Blanco, Brenda Natzke, Linda Nguyen

**Affiliations:** 1 Office of the Assistant Secretary for Planning & Evaluation, U.S. Department of Health and Human Services, 200 Independence Ave SW, Washington, DC 20543 USA; 2 Mathematica, 1100 First Street, NE, 12th Floor, Washington, DC 20002-4221 USA

**Keywords:** Neonatal abstinence syndrome, Substance use, Prenatal

## Abstract

**Introduction:**

Estimating Neonatal Abstinence Syndrome (NAS) and prenatal substance exposure rates in Medicaid can help target program efforts to improve access to services.

**Methods:**

The data for this study was extracted from the 2016–2020 Transformed Medicaid Statistical Information System (T-MSIS) Analytic Files (TAF) Research Identifiable Files (RIF) and included infants born between January 1, 2016 and December 31, 2020 with a either a NAS diagnosis or prenatal substance exposure.

**Results:**

Between 2016 and 2020, the estimated national rate of NAS experienced a 18% decline, while the estimated national rate of prenatal substance exposure experienced a 3.6% increase. At the state level in 2020, the NAS rate ranged from 3.2 per 1000 births (Hawaii) to 68.0 per 1000 births (West Virginia). Between 2016 and 2020, 28 states experienced a decline in NAS births and 20 states had an increase in NAS rates. In 2020, the lowest prenatal substance exposure rate was observed in New Jersey (9.9 per 1000 births) and the highest in West Virginia (88.1 per 1000 births). Between 2016 and 2020, 38 states experienced an increase in the rate of prenatal substance exposure and 10 states experienced a decline.

**Discussion:**

Estimated rate of NAS has declined nationally, but rate of prenatal substance exposure has increased, with considerable state-level variation. The reported increase in prenatal substance exposure in the majority of US states (38) suggest that substances other than opioids are influencing this trend. Medicaid-led initiatives can be used to identify women with substance use and connect them to services.

**Supplementary Information:**

The online version contains supplementary material available at 10.1007/s10995-023-03670-z.

## Significance

*What is already known about the topic?* Neonatal Abstinence Syndrome (NAS) and prenatal substance exposure are significant risk factors for poor neurodevelopmental and mental health outcomes in early childhood. NAS birth rates have been increasing in the US since 2000 and the majority of NAS births are covered by Medicaid.

*What this article adds?* This article estimates national and state-level prenatal substance exposure and NAS rates among Medicaid-covered infants born between 2016-2020 using data from the Transformed Medicaid Statistical Information System. This is the first study using post-2017 data to estimate national NAS rates. The findings can inform future federal and state policy efforts to improve access to screening, diagnosis and treatment among pregnant women with substance use disorder and infants with NAS.

**Supplementary Information:**

The online version contains supplementary material available at 10.1007/s10995-023-03670-z.

## Introduction

Neonatal abstinence syndrome (NAS) is a group of physiologic and neurobehavioral signs of withdrawal that may occur in a newborn who was exposed to psychotropic substances in utero (SAMHSA, 2018). Infants with NAS have higher emergency department and rehospitalization rates than infants without NAS (Ali et al., 2020) and are more likely to meet criteria for educational disability at school age (Fill et al., 2018). NAS births rates in the US started increasing in 2000 (Patrick et al., 2012) and nearly doubled between 2010 and 2017 (Hirai et al., 2021).

More recent hospital discharge data show that there was a decline in the national NAS rate between 2017 and 2018 (HCUP, 2021), and one study from Nevada showed a slight decline in NAS incidence rates between 2016 and 2018 (Batra et al., 2021).There is dearth of studies using post-2018 data to analyze national trends in NAS rates, whether the decline in NAS rate has continued, and how state estimates relate to the national trends post-2018. This is an important omission in the literature as the worsening of the substance use disorder (SUD) crisis during COVID-19 (CDC, 2023) and the increase in polysubstance use (Cicero et al., 2020) have resulted in substantial shifts in opioid and non-opioid drug use trends. This has potential implications for not only health care delivery, but also substance use prevention and treatment.

The shift in non-opioid drug use, such as methamphetamine, other psychostimulants, and alcohol, highlights the importance of estimating trends in prenatal substance exposure, along with NAS—something that has received limited attention in the literature. In addition, Medicaid is a primary payer for NAS-related births in the US, covering more than 80% of all NAS births with a yearly cost of $462 million in 2014 (Winkelman et al., 2018). While NAS and maternal opioid-related diagnosis rates can vary widely between states (Hirai et al., 2021), no study to date has estimated their state-to-state variation in Medicaid with national, population-level data.

This study aims to estimate the NAS and prenatal substance exposure birth rate among Medicaid covered births using 2016–2020 claims data by state and nationwide in order to inform future national and state policy-planning aimed at this population. Prenatal substance exposure was estimated in addition to NAS, as NAS coding has been typically associated with prenatal opioid exposure in claims data (Jilani et al., 2022), while neonatal withdrawal symptoms can be caused by a number of substances (SAMHSA 2018). Estimation of both NAS rates and prenatal substance exposure rates in Medicaid covered births are important for federal policymakers in designing better targeted programs nationwide and for state policymakers in evaluating outcomes from state prevention and treatment efforts.

## Methods

The data for this study were extracted from the 2016–2020 Transformed Medicaid Statistical Information System (T-MSIS) Analytic Files (TAF) Research Identifiable Files (RIF). This analysis includes children with an enrollment record in the TAF RIF and whose births were covered by Medicaid with a date of birth between January 1, 2016 and December 31, 2020. We identified newborn births using a select set of diagnosis codes on claims (Online Appendix A). Some states have serious data quality issues, making the data unusable. To assess data quality, we used information on measures featured in the T-MSIS Data Quality Atlas (https://www.medicaid.gov/dq-atlas/welcome) excluding any measures deemed unusable. The final analytic sample includes all Medicaid covered birth from 47 states and the District of Columbia. Data for three of the US states, Tennessee, Rhode Island, and Maryland, was not included in the study sample because of data quality limitations. The sample included all Medicaid covered births (between 1.6 million and 1.4 million) out of which, 20,004–25,509 infants had a NAS diagnosis and 44,145–48,466 infants had a prenatal substance exposure diagnosis (please see online Appendix Table B for a year-by-year breakdown of the trends).

A descriptive analysis was conducted to estimate the number and rates of newborn Medicaid beneficiaries with prenatal substance exposure and NAS diagnosis by state and birth year expressed as per 1,000 Medicaid-covered births. P96.1 was used to identify children with NAS diagnosis based on its high positive predictive value (Elmore et al., 2020); and because it is the most commonly used ICD-10 code for NAS surveillance by states (Chiang et al., 2019). Prenatal substance exposure was defined as the use of illegal and legal substances during pregnancy based on select ICD-10 codes (see online Appendix C). These codes were included since neonatal withdrawal can be caused by a number of substances and given the connection between maternal drug use and neonatal withdrawal symptoms. The TMSIS TAF data include all Medicaid covered births in the US and as such represent a census of all those with NAS or prenatal substance exposure; thus, a test of statistical difference was not conducted as any year-to-year difference will be meaningful.

## Results

**Fig. 1:**
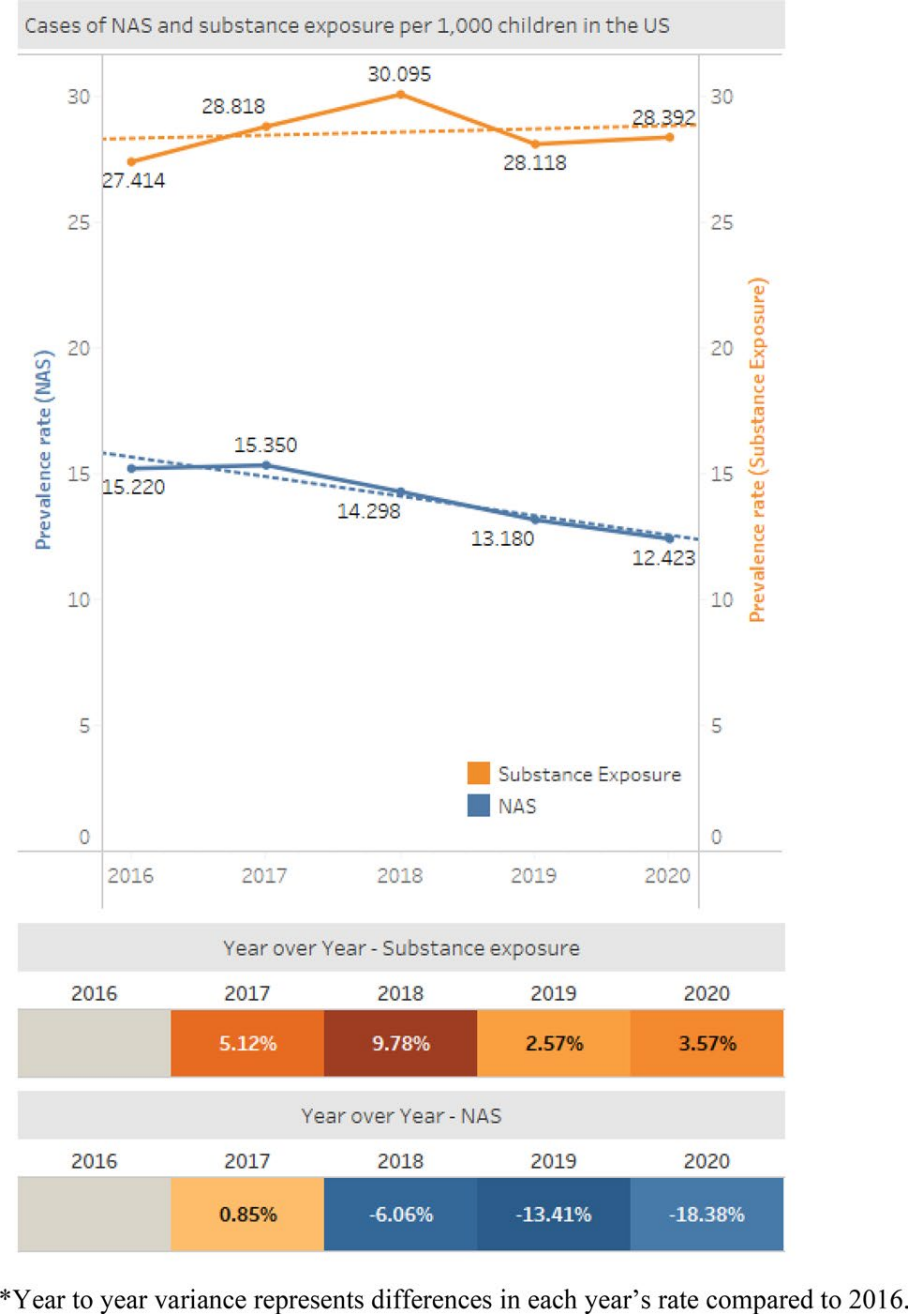
National trends in NAS and prenatal substance exposure. Prenatal substance exposure increased from 27.414 substance-exposed newborns per 1000 births in 2016 to 28.392 in 2020. NAS rates decreased from 15.220 infants with NAS diagnosis per 1000 births in 2016 to 12.423 in 2020. Year to year variance represents differences in each year’s rate compared to 2016

Between 2016 and 2020, the national rate of NAS experienced a 18% decline, from 15.2 per 1000 births to 12.4 per 1000 Medicaid births (Fig. 1). The national rate of prenatal substance exposure experienced a 3.6% increase between 2016 and 2020, going from 27.4 per 1000 births in 2016, to 30.1 per 1000 Medicaid births in 2018, and then to 28.4 per 1000 Medicaid births in 2020.

**Fig. 2:**
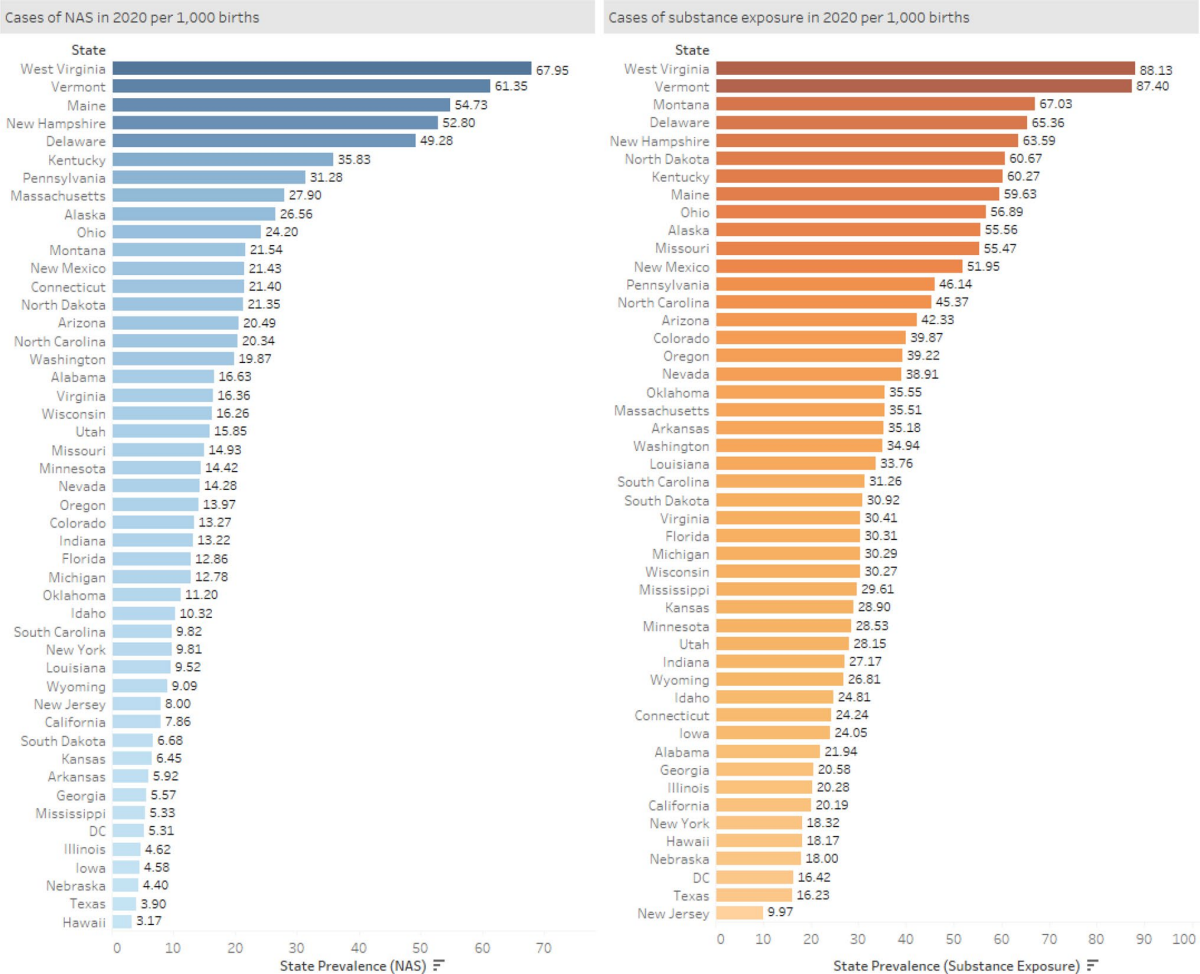
State NAS and prenatal substance exposure estimates, 2020. In 2020, the NAS rate ranged from 3.17 per 1000 births in the state of Hawaii to 67.95 per 1000 births in West Virginia. The lowest prenatal substance exposure rate ranged from 9.97 per 1000 births in New Jersey and to 88.13 per 1000 births in West Virginia

**Fig. 3:**
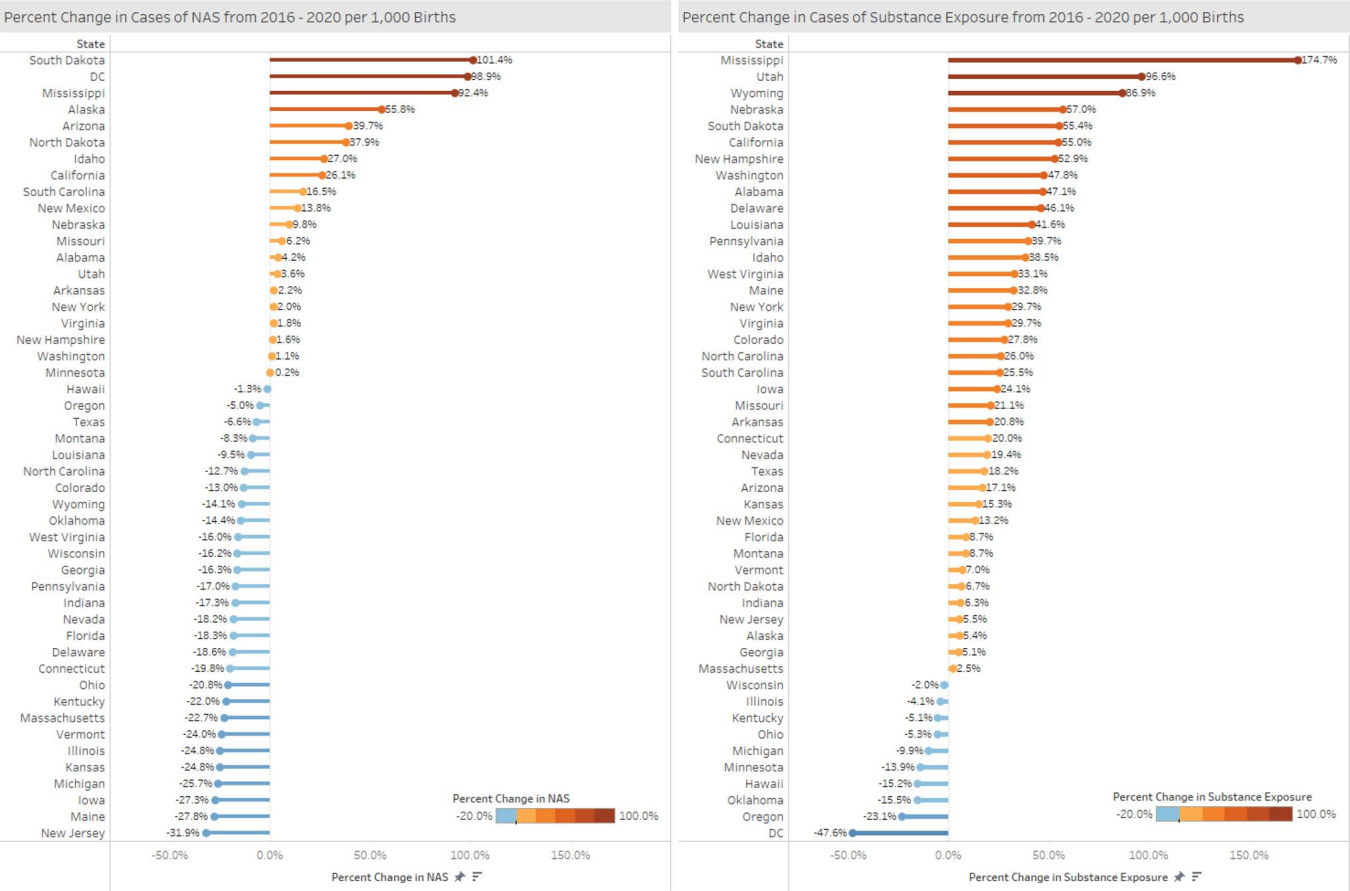
Percent change in NAS and prenatal substance exposure rates, 2020–2016: geographic distribution. Between 2016 and 2020, 28 states experienced a decline in NAS birth rate, ranging between − 31.9% (New Jersey) and − 1% (Hawaii). 38 states experienced an increase in the rate of prenatal substance exposure, ranging from 2.5% (Massachusetts) to 174.7% (Mississippi)

**Fig. 4:**
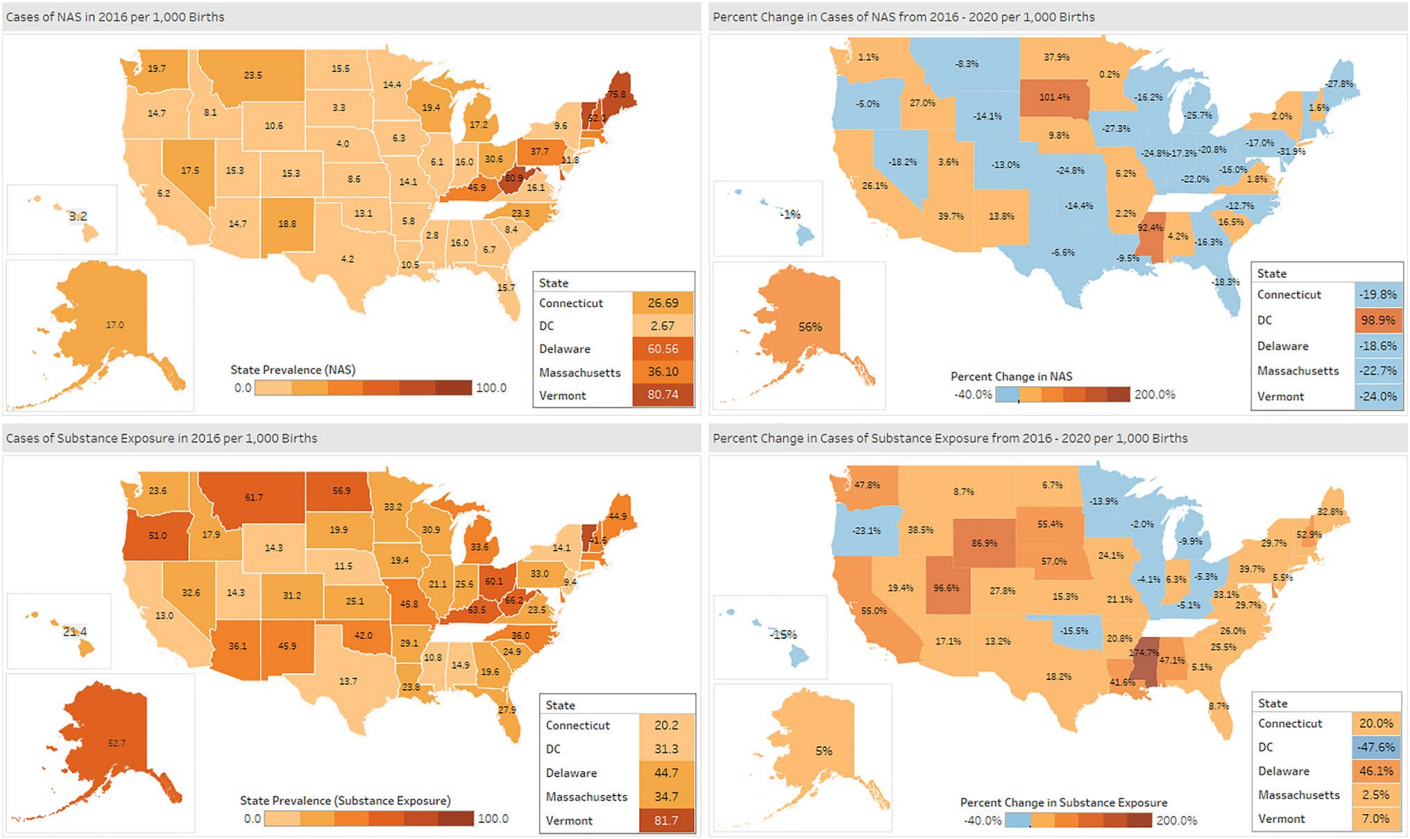
Percent change in NAS and prenatal substance exposure rates, 2020–2016: list of states. Between 2016 and 2020, 10 states had NAS rate increases above 10%. Three states had close to 100% increase in their prenatal substance exposure rates by 2020- South Dakota (101.4%), DC (98.9%) and Mississippi (92.4%). 29 states had more than 10% increase in their prenatal substance exposure rate, and three states had close to or above 100% increases (Wyoming, Utah, Missisippi)

At the state level in 2020, the NAS rate ranged from 3.2 per 1000 births in the state of Hawaii to 68.0 per 1000 births in West Virginia (Fig. 2). In 2020, the lowest prenatal substance exposure rate was observed in New Jersey (9.9 per 1000 births) and the highest in West Virginia (88.1 per 1000 births). Between 2016 and 2020, 28 states experienced a decline in NAS births, with 23 states of them having 10–30% decline (Fig. 3). Twenty states had an increase in NAS rates between 2016 and 2020, with Minnesota experiencing the smallest (0.2%) and South Dakota experiencing the largest increase (101.4%) (Fig. 4). Ten states had NAS rate increases above 10%; with three of those states having to 100% increase in their prenatal substance exposure rates- South Dakota (101.4%), DC (98.9%) and Mississippi (92.4%) (Fig. 4).

Between 2016 and 2020, 38 states experienced an increase in the rate of prenatal substance exposure and 10 states experienced a decline, compared to 2016 levels (Fig. 3). Massachusetts experienced the smallest increase (2.5%) and Mississippi experienced the largest increase (174.7%). Overall 29 states had more than 10% increase in their prenatal substance exposure rate (Fig. 3). Two states had close to 100% increase, Wyoming (86.9%) and Utah (96.6%), and Mississippi had a 174.7% in its prenatal substance exposure rate by 2020.

**Fig. 5:**
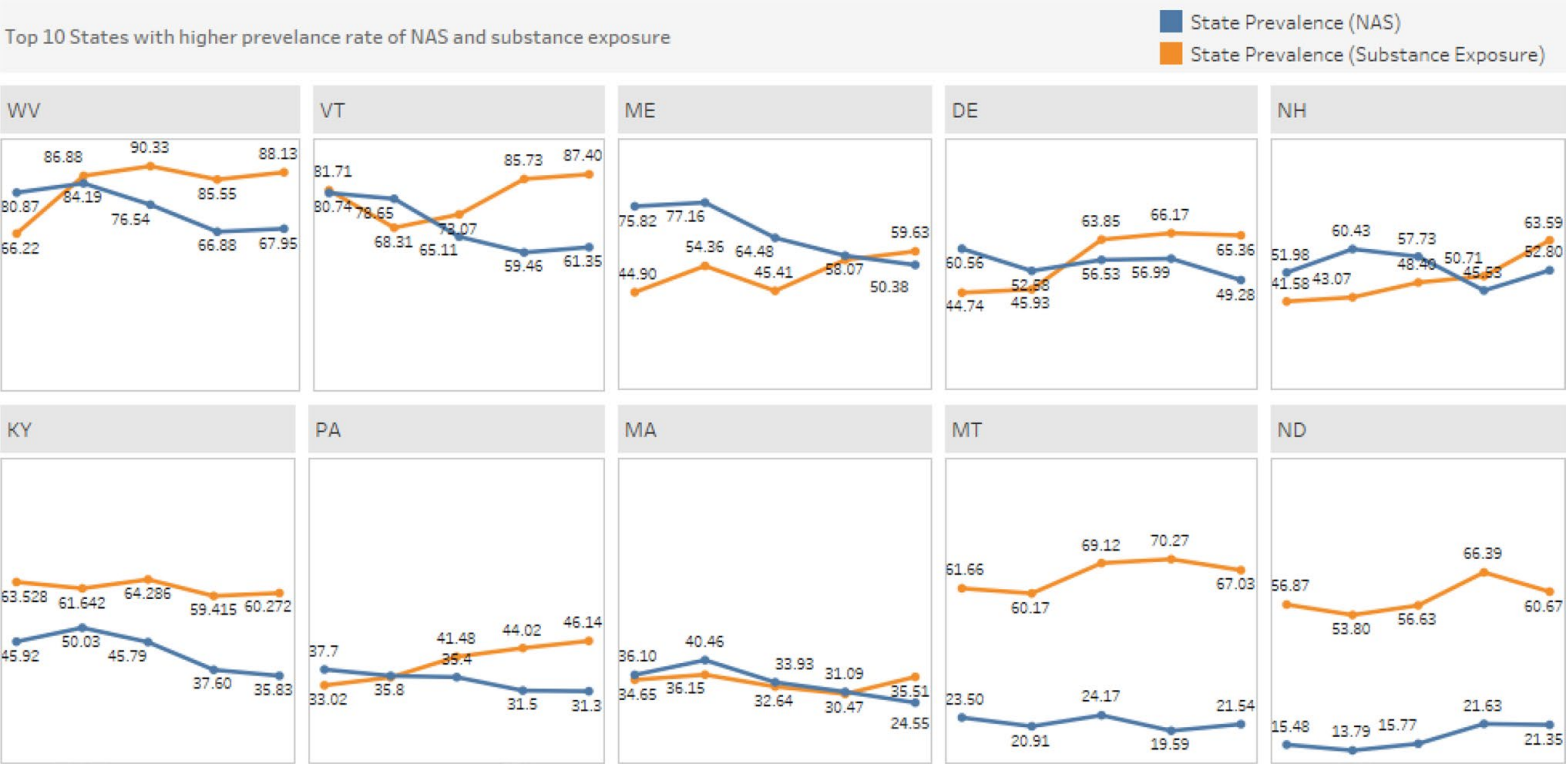
State trends in 10 states with the highest NAS and prenatal substance exposure rates, 2020–2016. Among the 10 states with the highest NAS and prenatal substance exposure rates, 9 states had both decreasing NAS rates and increasing prenatal substance exposure rates with the exception of New Hampshire and Kentucky. In New Hampshire both prenatal substance exposure rate (from 41.58 in 2016 to 63.59 in 2020) and NAS rate (from 51.98 in 2016 to 52.80 in 2020) increased while Kentucky had declines in both prenatal substance exposure rates (from 63.52 in 2016 to 60.27 in 2020) and NAS rates (from 42.92 in 2016 to 35.83 in 2020)

When looking at trends in NAS and prenatal substance exposure in the 10 states with the highest NAS and prenatal substance exposure rates (Fig. 5), we see that the rate of prenatal substance exposure has had an upward trend and was higher compared to NAS for most states. One exception was New Hampshire for which both prenatal substance exposure and NAS rate increased and Kentucky, which had declines in both rates.

## Discussion

In the course of five years, between 2016 and 2020, NAS rates recorded in Medicaid claims decreased nationally, while prenatal substance exposure increased. More than half of the states in the US (28) have a declining NAS rate. Given that the majority of the states in the study (38) reported increases in prenatal substance exposure, this could be an indicator that substances other than opioids are influencing this trend, particularly those not directly linked to NAS. It is possible that the previously reported decreases in opioid use since 2017 are contributing to the declining NAS trends (SAMHSA, 2022). However, the increase in prenatal substance exposure that this study finds is concerning and coincides with previously reported increases in prenatal marijuana, alcohol and other illicit drug use (SAMHSA, 2022; Volkow et al., 2019) and binge drinking among pregnant women (Howard et al., 2022). Thus, it seems likely that the upward trend in prenatal substance exposure in Medicaid that this study finds is connected to larger trends in substance use among pregnant women in the US.

The reasons behind the decline in NAS rates are likely multifaceted as NAS birth rates are influenced by social and economic factors, availability of health care (Patrick et al., 2019), as well as local and state policies. For example, punitive state policies that criminalized substance use during pregnancy, consider it grounds for civil commitment, or consider it to be child abuse or neglect, have been found to be associated with higher odds of NAS and can deter women from accessing substance use disorder (SUD) services (Faherty et al., 2019; Atkins et al., 2020; Kozhimannil et al., 2019). Punitive policies also vary from state to state which could impact the access to treatment and NAS/prenatal substance exposure identification. Variation in clinical case definitions and used diagnosis codes is also likely affecting the differences in NAS rates among states (Chiang et al., 2019). Finally, use of medications for opioid use disorder (MOUD) by the mother during pregnancy may also cause withdrawal symptoms, depending on the medication used (Suarez et al., 2022). MOUD prescribing patterns have been found to vary by state (Clemens-Cope et al. 2019) and could influence mother’s chance of using MOUD. MOUD is highly recommended during pregnancy because it reduces adverse outcomes for the mom and the baby (SAMHSA, 2018).

These trends in NAS and prenatal substance exposure overlap with a number of policy developments since 2015 aimed towards addressing the opioid crisis. For example, in 2015 the US Department of Health & Human Services (HHS) announced a strategy to curb opioid and heroin overdose and mortality which sought to improve safe and appropriate opioid prescribing, expand access to overdose reversal medications, and expand use of medication for opioid use disorder (HHS, 2016). The following year, the Centers for Disease Control and Prevention (CDC) released a guideline for prescribing opioids for chronic pain management (Dowell et al., 2016). The guideline required physicians (for the first time) to weigh the risks and benefits associated with opioid use before prescribing and had specific recommendations for pregnant women. While the impact of the guidelines on pregnant women alone has not been evaluated, past research has found association between the CDC guidelines and the reduced opioid prescribing 2017–2018 (Townsend et al., 2021; Sutherland et al., 2021). In addition, one study has suggested that opioid prescribing might be higher in counties with high NAS rates (Fingar et al., 2021). It is possible that the extensive policy engagements at both the federal and state level might have contributed to the decline in national NAS rates observed in the current analysis.

States in the Northeast (VT, ME, NH) and some of the Mid-Atlantic states (WV, DE) have continued to have overall the highest NAS rates in the US during the study period. There were no regional patterns in the prenatal substance exposure: every US region had states with reported high increases in prenatal substance exposure, except to some degree the Midwest where the majority (but not all) states reported declines. This variation can be an indicator of state-specific policies and availability of prevention and treatment programs. The increase can also be related to regional drug supply patterns and increase in the use of synthetic opioids and non-opioid substances, including alcohol (Ciccero et al., 2020).

The between state variations clearly show which states have made strides in addressing NAS and prenatal substance exposure and which need additional resources and policy action to address either or both. Policymakers at the state level can use the findings of this analysis to further investigate what type of substance use in particular is driving these trends. Researchers can investigate contextual factors that have impacted reduction in NAS and increase in prenatal substance exposure in order to understand how local factors and policy approaches can be adjusted to be most effective. Screening and treatment approaches would need to be adapted as therapeutic approaches differ between opioids, stimulants, tobacco, or alcohol.

Continued investment at the federal level is important. Given the increased use of other substances such as alcohol and stimulants among pregnant women (Howard et al, 2022; Bruzelius et al. 2022), addressing and preventing opioid use should be coupled with improving access to treatment of other substances in addition to opioids. Medicaid-led initiatives such as the MOM innovation model can be used to identify people with substance use needs, provide coordinated care, and adjust treatment to the needs of the local community. Sustained effort to incentivize adoption of innovative payment models among maternity care providers and primary care providers can help improve screening for and connect women to SUD services (Seibert et al., 2022). The 12-month postpartum coverage extension in Medicaid, made permanent with the 2022 Consolidated Appropriations Act, provides an opportunity for providers to engage with women postpartum, and facilitate access to needed medication and/or psychosocial treatment.

To our knowledge, this is the first study that shows a trend in declining NAS rates using the most current national Medicaid data (2016–2020). Although the study is limited to Medicaid covered births, two factors make it possible that these findings represent an overall national trend. First, the trends found here are consistent with the decline in NAS rates between 2017 and 2018 that were estimated using national hospital discharge data (HCUP, 2021). Secondly, 84% of NAS births are covered by Medicaid (Hirai et al., 2021), and therefore Medicaid-covered NAS births are a major driver of national trends. Strengths of our study include using a nationwide claims database and presenting individual state estimates for 47 states and the District of Columbia. Since the analysis used 2016–2020 data, COVID-19 restrictions may have affected use of screening and diagnostic services and potentially impacted NAS identification in 2020; however, given the 2020 data is part of an overall trend of declining NAS rates of four years in a row the presenting data likely accurately mirrors the NAS birth rate. Our study is using Medicaid administrative data which depends on the accuracy of coding and is a conservative estimate; previous research has found that claims data-based analysis underestimate NAS rates (Elmore et al., 2020, Ernst & Makkar, 2018). In addition, for certain substance (e.g. alcohol) prenatal substance exposure codes may be assigned to patients based on self-report which can underestimate the true prevalence.

Addressing prenatal substance exposure is an important public health priority, as substance exposure, along with mental health conditions, is a leading underlying cause of maternal mortality (Trost et al., 2022) and more than two-thirds of women with pregnancy-related mental health deaths have had substance use (Trost et al., 2021). Access to SUD treatment can help keep families together and parents in treatment (Ali et al., 2022),with the best outcomes for both pregnant and postpartum mothers and with NAS. Dyadic treatment approaches for both the mother and the infant and family centered care are recommended for addressing NAS (Jansson et al., 2019, Mossabeb et al., 2021). Universal substance screening among reproductive age women coupled with brief intervention and referral to treatment has the potential to reduce the burden of substance use in pregnancy (Wright et al., 2016). This together with improved access to dyadic care and family-centered treatment can help address the urgent need for services among this population.

## Supplementary Information

Below is the link to the electronic supplementary material.

Supplementary file1 (DOCX 19 KB)

## Data Availability

Proprietary data available from Centers for Medicare and Medicaid Services with Data Use Agreement.

## Code Availability (software application or custom code)

The coding for this analysis is available upon request.
