# Correction: Prenatal Substance Exposure and Neonatal Abstinence Syndrome: State Estimates from the 2016–2020 Transformed Medicaid Statistical Information System

**DOI:** 10.1007/s10995-023-03763-9

**Published:** 2023-07-17

**Authors:** Kristina D. West, Mir M. Ali, Martin Blanco, Brenda Natzke, Linda Nguyen

**Affiliations:** 1 Office of the Assistant Secretary for Planning & Evaluation, U.S. Department of Health and Human Services, 200 Independence Ave SW, 20543 Washington, DC USA; 2 Mathematica, 1100 First Street, NE, 12th Floor, 20002‑4221 Washington, DC USA

**Correction to: Maternal and Child Health Journal**

10.1007/s10995-023-03670-z

The original version of this article unfortunately contained a typo in article title.

In title, the word “Medical” should be “Medicaid”.

The correct title is: Prenatal Substance Exposure and Neonatal Abstinence Syndrome: State Estimates from the 2016–2020 Transformed Medicaid Statistical Information System.

The original article has been corrected.

