# Supplementary material for: Prenatal Substance Exposure and Neonatal Abstinence Syndrome: State Estimates from the 2016–2020 Transformed Medicaid Statistical Information System
Source: Matern Child Health J. 2023 May 23;27(Suppl 1):14–22. doi: 10.1007/s10995-023-03670-z (PMC10204012; doi:10.1007/s10995-023-03670-z)
Supplement: Supplementary file 1 — Supplementary file1 (DOCX 19 KB) [file 10995_2023_3670_MOESM1_ESM.docx]

**Appendix A**

| **ICD-10-CM Code** | **Definition** |
| --- | --- |
| Z3800 | Single liveborn infant, delivered vaginally |
| Z3801 | Single liveborn infant, delivered by cesarean |
| Z381 | Single liveborn infant, born outside hospital |
| Z382 | Single liveborn infant, unspecified as to place of birth |
| Z3830 | Twin liveborn infant, delivered vaginally |
| Z3831 | Twin liveborn infant, delivered by cesarean |
| Z384 | Twin liveborn infant, born outside hospital |
| Z385 | Twin liveborn infant, unspecified as to place of birth |
| Z3861 | Triplet liveborn infant, delivered vaginally |
| Z3862 | Triplet liveborn infant, delivered by cesarean |
| Z3863 | Quadruplet liveborn infant, delivered vaginally |
| Z3864 | Quadruplet liveborn infant, delivered by cesarean |
| Z3865 | Quintuplet liveborn infant, delivered vaginally |
| Z3866 | Quintuplet liveborn infant, delivered by cesarean |
| Z3868 | Other multiple liveborn infant, delivered vaginally |
| Z3869 | Other multiple liveborn infant, delivered by cesarean |
| Z387 | Other multiple liveborn infant, born outside hospital |
| Z388 | Other multiple liveborn infant, unspecified as to place of birth |

**Appendix B**

Number of children with NAS or PSE by year of data collection in TMSIS TAF Files

|  | **2016** | **2017** | **2018** | **2019** | **2020** |
| --- | --- | --- | --- | --- | --- |
| Number of children with NAS diagnosis | 24,509 | 24,729 | 23,035 | 21,223 | 20,004 |
| Number of children with prenatal substance exposure | 44,145 | 46,406 | 48,466 | 45,278 | 45,720 |
| Total number of Medicaid covered births | 1,610,301 | 1,587,626 | 1,541,810 | 1,509,597 | 1,459,422 |

**Appendix C**

| **ICD-10-CM Code** | **Definition** |
| --- | --- |
| P04.1A | Newborn affected by maternal use of anxiolytics |
| P04.13 | Newborn affected by maternal use of anticonvulsants |
| P04.14 | [Newborn affected by maternal use of opiates](https://www.aapc.com/codes/icd-10-codes/P04.14) |
| P04.15 | [Newborn affected by maternal use of antidepressants](https://www.aapc.com/codes/icd-10-codes/P04.15) |
| P04.16 | [Newborn affected by maternal use of amphetamines](https://www.aapc.com/codes/icd-10-codes/P04.16) |
| P04.17 | Newborn affected by maternal use of sedative hypnotics |
| P04.2 | [Newborn affected by maternal use of tobacco](https://www.aapc.com/codes/icd-10-codes/P04.2) |
| P04.3 | Newborn affected by maternal use of alcohol |
| P04.4 | Newborn affected by cocaine, hallucinogens and unspecified drugs of addiction |
| P04.81 | [Newborn affected by maternal use of cannabis](https://www.aapc.com/codes/icd-10-codes/P04.81) |
